# Impact of NaHCO_3_/Na_2_CO_3_ Buffer Reagent on Mitigating the Corrosion of C110 Steel in Water-Based Annulus Protection Fluid at Ultrahigh Temperature

**DOI:** 10.3390/ma18071668

**Published:** 2025-04-05

**Authors:** Zhi Zhang, Mifeng Zhao, Yan Li, Junfeng Xie, Wenwen Song, Juantao Zhang, Mengkai Wang, Jie Zhou, Yuan Wang, Xiaowei Lei, Danping Li

**Affiliations:** 1 R&D Center for Ultra-Deep Complex Reservoir Exploration and Development, China National Petroleum Corporation (CNPC), Korla 841000, China; zhangz-tlm@petrochina.com.cn (Z.Z.); zhaomf-tlm@petrochina.com.cn (M.Z.); liyan5-tlm@petrochina.com.cn (Y.L.); xjf-tlm@petrochina.com.cn (J.X.); songww-tlm@petrochina.com.cn (W.S.); 2 Engineering Research Center for Ultra-Deep Complex Reservoir Exploration and Development, Xinjiang Uygur Autonomous Region, Korla 841000, China; 3 Xinjiang Key Laboratory of Ultra-Deep Oil and Gas, Korla 841000, China; 4 Tarim Oilfield Company, PetroChina Company Limited, Korla 841000, China; 5 Tubular Goods Research Institute of China National Petroleum Corporation (CNPC), Xi’an 710077, China; zhangjuantao@cnpc.com.cn (J.Z.); wangmk@cnpc.com.cn (M.W.); zhoujie06@cnpc.com.cn (J.Z.); wangyuan008@cnpc.com.cn (Y.W.); 6 School of Physical Science and Technology, Northwestern Polytechnical University, Xi’an 710072, China; xiaowei_lei@nwpu.edu.cn

**Keywords:** C110 steel, annulus protection fluid, buffer solution, corrosion, inhibition

## Abstract

The drilling of ultradeep oil wells brings many challenges to the downhole tubular materials, where corrosion induced by halide annulus protection fluid is one major problem. In this work, the Na_2_CO_3_/NaHCO_3_ buffer system is employed to mitigate the corrosion of C110 steel in NaBr annulus protection fluid at 220 °C. Weight loss tests, corrosion morphologies characterizations, and electrochemical measurements were used to investigate the inhibition effect. X-ray diffraction and X-ray photo-electron spectroscopy were employed to analyze the surface phase compositions. It is found that the Na_2_CO_3_/NaHCO_3_ buffer reagents effectively inhibit the corrosion of C110 steel, and the inhibition efficiency can reach 96.1%. The higher pH leads to the better inhibition performance, and, particularly, the buffer system is more effective in the corrosion environment of greater aggressivity. Without buffer reagents, the steel substrate is subjected to higher degree of uniform etching and pitting corrosion due to the formation of loose and porous corrosion products. In contrast, the addition of buffer reagents facilitates the formation of thinner but denser and more protective Fe_3_O_4_ passive film, contributing the high corrosion inhibition efficiency. Our work paves the way for the safe service of NaBr annulus protection fluid at 220 °C in ultradeep oil wells.

## 1. Introduction

With the continuous growth of global energy demand, the exploration and development of oil and gas resources are expanding to deeper and more complex fields [1,2,3,4]. Against this backdrop, the drilling technology of 10,000 m deep wells in China has developed rapidly and become a key means to break through the traditional oil and gas exploration boundaries. However, the drilling of 10,000 m deep wells is facing unprecedented challenges, among which the ultrahigh temperature is particularly prominent [5,6]. In the extreme geological environment such as the Tarim Basin, the downhole temperature can reach 220 °C, which necessitates extremely strict requirements for various materials and equipment required for drilling operations.

As an indispensable part in the drilling process of 10,000 m deep wells, the performance of annulus protection fluids is directly related to the safety and efficiency of drilling operations [7,8,9,10,11]. In ultrahigh temperature environments, annulus protection fluids need to have good thermal stability, high-temperature oxidation resistance, and excellent lubrication properties to ensure effective protection of wellbore walls, prevent formation damage, and improve drilling efficiency under high temperature and pressure conditions [12,13]. In particular, annulus protection fluids need to be less corrosive to reduce the negative impact on the well material [14,15]. Therefore, the research and development of annulus protection fluids adapted to ultrahigh temperature service conditions has become an important issue to be solved urgently in the field of oil and gas exploration.

Commonly used annulus protection fluids can be divided into oil-based and water-based [7]. Oil-based ones have low density and insufficient high-temperature stability [16]; therefore, it is clear that oil-based annulus protection fluids cannot be applied to 10,000 m deep wells. For water-based annulus protection fluid, the most common type is halogen salts [17], such as NaBr aqueous solution which has high density and excellent high-temperature stability. However, due to the concentrated halide ions and favored electrochemical reactions with increasing temperature [18], halogen salts are very corrosive to the tubular materials at high temperatures, remaining a tough challenge up to now. In order to reduce the corrosion of halogen annulus protection fluid, it is crucial and urgent to develop inhibitors to mitigate the high-temperature halogen salt corrosion of the ultradeep well tubes operated at 220 °C.

Film-forming inhibitors or organic additives are widely explored in annulus fluid environments [1]. For instance, Obot et al. [19] reported that imidazoline inhibitors have fairly good inhibition efficiency in brine solution. Nevertheless, due to the thermal decomposition and the generation of oxide film at temperatures above 150 °C, the inhibition efficiency notably declines. In contrast, the inhibition performances of amine-based inhibitors, another category of film-forming inhibitors, are even worse because they are negatively affected by the growth of FeCO_3_ film on the steel surface at temperatures below 150 °C. Olivo et al. [20] analyzed the inhibition effect of alkyl compounds for the protection of mild steel in a CO_2_ corrosion environment. They found that the tail length of alkyl molecules modifies the activation energy of the electrochemical process, and a longer tail renders higher mitigation efficiency, but is only effective at low temperatures. As can be seen from the literature, organic inhibitors are clearly not applicable at 220 °C due to the destruction of organic molecular structure at high temperature.

According to Beverskog and Puigdomenech [21], the Pourbaix diagram of Fe at 200–300 °C shows a stable passive region in the pH range of 6.5–10, inclining to form Fe_2_O_3_ (at higher potential) and Fe_3_O_4_ (at lower potential) to prohibit anodic dissolution. In particular, the passive region of Fe in the Pourbaix diagram shifts to the negative potential direction with increased pH (towards alkaline condition) [21,22], which aligns well with the decrease in electrode potential of Fe at elevated temperatures. Inspired by this, we presume that tuning the pH value of halogen annulus protection fluid to 9–10 by using alkaline species may work in the present service condition. Meanwhile, considering the high concentration of aggressive halide ions, the passivation state of Fe needs to be very stable, which requires abundant oxygen-ion sources provided by the inhibitor, i.e., containing high content of oxygen-rich anion species. In addition, the pH also has to be stable to avoid deterioration of the passive film, indicating that the buffering system should be appropriate.

Based on the above thoughts, we employ the Na_2_CO_3_/NaHCO_3_ buffer system in this study, which has not been reported before in the condition of halide-concentrated annulus protection fluid at 220 °C. By adjusting the pH value of the fluid to 9–10, it is anticipated that the buffer system can provide a good inhibition effect on the corrosion of carbon steel. We conducted a weight loss test, corrosion morphology characterizations, and electrochemical measurements to investigate the inhibition effect. X-ray diffraction (XRD) and X-ray photoelectron spectroscopy (XPS) were used to analyze the surface phase composition. The corrosion inhibition mechanism of the Na_2_CO_3_/NaHCO_3_ buffer system is discussed in detail. Our work not only improves the high-temperature service safety of tubular materials but also provides a strong support for the development of the drilling technology of 10,000 m ultra-deep oil wells.

## 2. Materials and Methods

The tubular material used in the present study is C110 carbon steel, and its chemical composition is given in Table 1. The microstructure of this steel is tempered sorbite, which is composed of ferrite and carbide fine particles. The samples were cut into coupons with the dimension of 50 × 10 × 3 mm. Prior to all the tests, the C110 samples were polished using the SiC sandpapers from 300 to 1200 grit, washed by deionized water and ethanol, and dried by a cool air drier.

The NaBr annulus protection fluid was prepared by adding 39.4 wt% NaBr into deionized water, obtaining a solution with a density of 1.4 g cm^−3^ and pH of 7.30. For the Na_2_CO_3_/NaHCO_3_ buffer solution, three different concentration ratios of Na_2_CO_3_:NaHCO_3_, namely, 2.3:1, 7.2:1, and 19.8:1, respectively, were used to adjust the NaBr annulus protection fluid into the pH values of 9.0, 9.5, and 10.0. The corresponding fractions of the buffer system in the solution are 0.64 wt%, 1.62 wt%, and 2.00 wt%, respectively.

For the weight loss test, three parallel coupons samples were used for each test. Immersion corrosion was conducted in the aforementioned solution at 220 °C for 7 and 30 days in an autoclave (total pressure 10 MPa by N_2_). Subsequently, the samples were taken out and cleaned with the solution of 3% HCl + 1% hexamethylenetetramine to remove the corrosion products. Afterward, the samples were washed with deionized water and ethanol and dried. The masses of samples were weighed before and after the immersion tests. The corrosion rate (*CR*) was calculated by using Equation (1) [7]:

(1) $$CR=87,600\;\frac{∆m}{\rho At}$$

where *CR* represents the corrosion rate each year (mm a^−1^), Δ*m* is the weight loss (g), *ρ* is density of the steel (7.86 g cm^−3^), *A* is the surface area (cm^2^), and *t* is immersion time. After immersion, the samples’ surfaces were characterized using scanning electron microscope (SEM, Tescan Vega 3 LMH, Tescan Co., Ltd., Brno—Kohoutovice, Czech Republic), energy dispersive spectrum (EDS, Oxford INCA X-ACT analyzer, Oxford Instruments Co., Ltd., Oxford, UK), XRD (Bruker D8 Advance, Bruker AXS Co., Ltd., Karlsruhe, Germany), and XPS (Kratos AXIS Ultra DLD analyzer, Al Kα, 1486.6 eV, Shimadzu Co., Ltd., Kyoto, Japan).

The electrochemical samples were cut into the dimensions of 10 × 10 × 3 mm, connected with a Cu wire and sealed by epoxy resin, with an exposure area of 10 × 10 mm^2^. The electrochemical measurements were carried out in the aforementioned solution using a Corrtest CS310M workstation (Wuhan Corrtest Instrument Co., Ltd., Wuhan, China) with Pt mesh as the counter electrode and a saturated calomel electrode (SCE) as the reference electrode. Before the test, the sample was placed into the cell at open circuit state for 30 min to stabilize the corrosion system. After that, the potentiodynamic polarization measurement was started from −0.15 V (vs. OCP) and swept positively with a rate of 0.5 mV s^−1^, and the test was terminated when the current density reached 10^−2^ A cm^−2^. All the electrochemical tests were repeated three times to ensure the reproducibility.

## 3. Results

### 3.1. Corrosion Performance in Deaerated Solution

**Figure 1:**
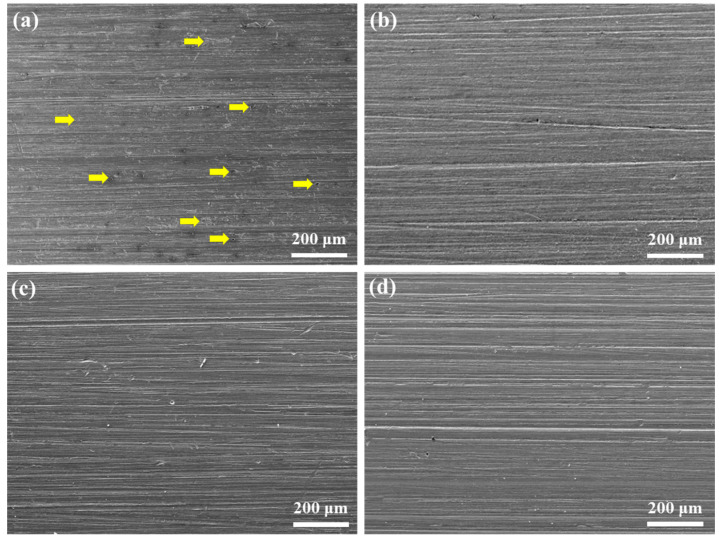
Surface morphologies of C110 steel after immersion corrosion at 220 °C for 7 days in deaerated NaBr solution without buffer reagents (**a**) and with buffer reagents adjusting the pH values to 9.0 (**b**), 9.5 (**c**), and 10.0 (**d**).

The corrosion mitigation effect of Na_2_CO_3_/NaHCO_3_ buffer solution is analyzed using the immersion corrosion test at 220 °C. The corrosion morphologies after immersion for 7 d and cleansing of the corrosion products are shown in Figure 1. In the absence of buffer reagents, many corrosion pits can be seen on the sample’s surface, and the whole surface is etched due to the accelerated dissolution effect of aggressive Br^−^ ions at 220 °C, as seen in Figure 1a. Since the C110 steel is composed of ferrite and carbide fine particles, the passivation process mainly occurs in the ferrite phase. However, according to the passivation theory, the carbides tend to weaken the stability of the passive film, inducing localized passivity breakdown and pitting corrosion. The addition of buffer reagents notably suppresses the dissolution, as evidenced by the clean surfaces in Figure 1b–d, where very few pits can be found and the abrasive scratches are clear. Specifically, when the pH = 9.0, the surface is slightly etched, while the other two samples with pH values of 9.5 and 10.0 are nearly not etched, indicating that the corrosion inhibition effect of Na_2_CO_3_/NaHCO_3_ buffer solution increases with the pH.

**Figure 2:**
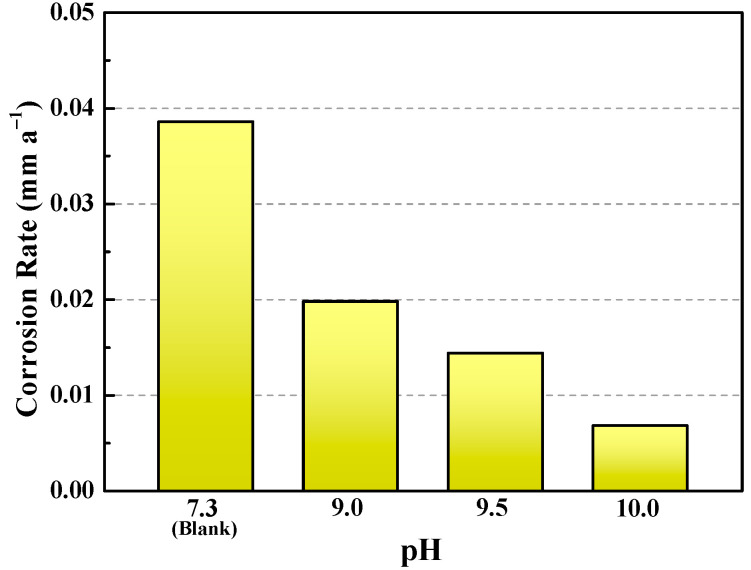
Corrosion rates of C110 steel after 7 days immersion at 220 °C in deaerated NaBr solution without buffer reagents (blank) and with buffer reagents (pH = 9.0, 9.5, and 10.0).

Figure 2 shows the corrosion rates of the C110 coupon samples after immersion corrosion at 220 °C for 7 d. For the blank test without buffer reagents, the corrosion rate is 0.0386 mm a^−1^, nearly two times greater than that of the pH 9.0 condition (0.0198 mm a^−1^). Further increasing the buffered pH values to 9.5 and 10.0, the corrosion rate gradually declines and reaches 0.0068 mm a^−1^ finally (pH = 10.0), under which the inhibition efficiency is 82.4%. The corrosion rate data agree well with the corrosion morphologies shown in Figure 1. Particularly, the high corrosion rate of the blank sample is partially attributed to pitting corrosion, which is more dangerous for tubular structures. In fact, we have conducted comparisons by using the inhibitor that is currently used in the domestic industry, i.e., the quinoline + zolazine based compound organic inhibitor. Based on our laboratory tests, the corrosion rate is 0.0430 mm a^−1^, which is about one order of magnitude (see Figure 2, pH = 10.0) greater than the Na_2_CO_3_/NaHCO_3_ inhibitor devised in our present work. More importantly, a lot of black suspended particles appeared in the solution for the organic inhibition system, which is due to the coking of organic species under 220 °C conditions, indicating that the organic inhibitors are not applicable in the ultra-high temperature environment.

**Figure 3:**
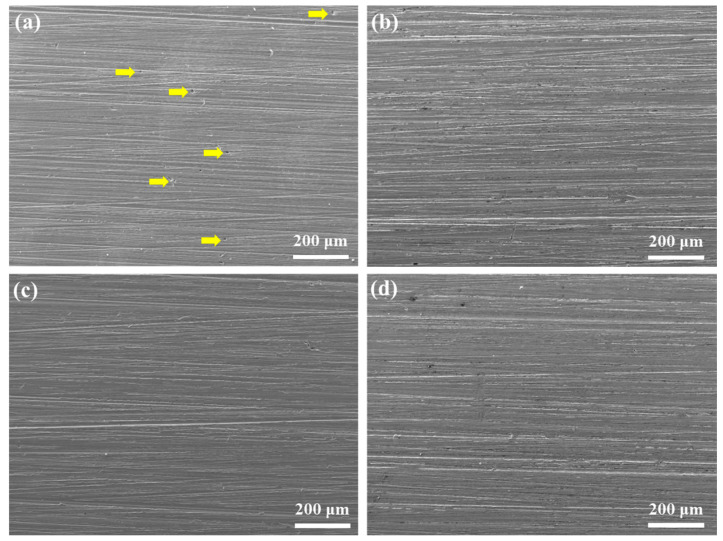
Surface morphologies of C110 steel after immersion corrosion at 220 °C for 30 days in deaerated NaBr solution without buffer reagents (**a**) and with buffer reagents adjusting the pH to 9.0 (**b**), 9.5 (**c**), and 10.0 (**d**).

The inhibition performance of Na_2_CO_3_/NaHCO_3_ buffer reagents is also investigated via the 30 d immersion test, and the corrosion morphologies are shown in Figure 3. Different from the 7 d immersion results, the extended immersion duration results in the etching of all the samples. Meanwhile, the blank sample shows many pitting corrosion sites, while other samples, in the presence of buffer reagents, exhibit general corrosion feature, and the corrosion degree declines with the increase in pH.

**Figure 4:**
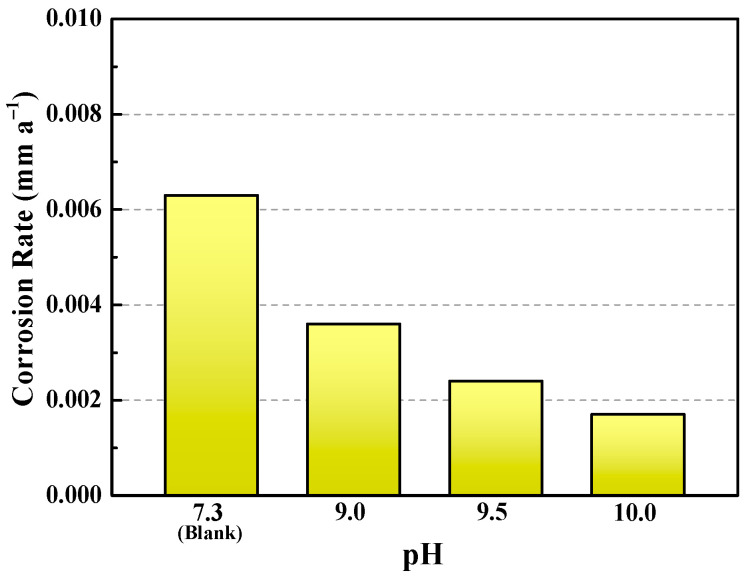
Corrosion rates of C110 steel after 30 days immersion at 220 °C in deaerated NaBr solution without buffer reagents (blank) and with buffer reagents (pH = 9.0, 9.5, and 10.0).

The corrosion rates of the samples in the absence and presence of buffer reagents are given in Figure 4. The corrosion rate gradually decreases with the elevated pH. For instance, the blank sample possesses the corrosion rate of 0.0063 mm a^−1^; however, when the pH is adjusted to 10.0, the corrosion rate drops to 0.0017 mm a^−1^, i.e., the inhibition efficiency can still maintain 73.1%. It should be noted that when the NaBr solution is in a deaerated state, the corrosion medium is relatively mild. As can be seen in Section 3.2, the Na_2_CO_3_/NaHCO_3_ buffer system shows much better protection ability in more aggressive environments containing O_2_, CO_2_, and H_2_S, which will be discussed later. Comparing these results with Figure 2, it is clear that the corrosion rate with a longer immersion time (30 d) is lower than that with a shorter immersion duration (7 d). This is ascribed to the formation of corrosion products which act as a barrier that hinders the corrosion reactions and, as the time extends, the bare steel is covered with thicker corrosion products and thus shows declined corrosion rate.

### 3.2. Corrosion Performance in Aerated Solution

The aforementioned experimental results are mainly focused on the deaerated solution, where the impact of dissolved oxygen is absent. In real oil-field operations, especially the annulus protection fluids of vast injection amount, the deaeration process may not be facile. Hence, it is necessary to evaluate the inhibition effect of the buffer system in an aerated corrosion environment. Below, we compare the corrosion behaviors of C110 steel without and with buffer reagents (pH adjusted to 10.0) in aerated NaBr solution.

**Figure 5:**
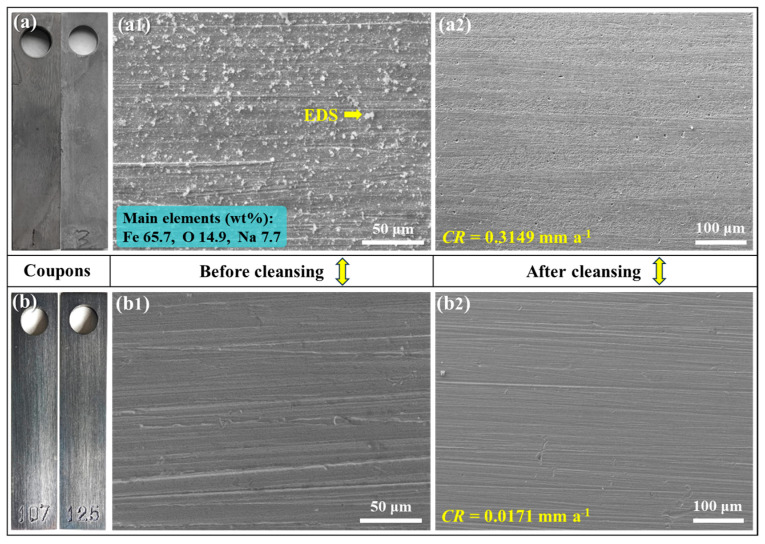
Surface morphologies of C110 steel after immersion corrosion at 220 °C for 30 days in aerated NaBr solution without buffer reagents (**a**,**a1**,**a2**) and with buffer reagents adjusting the pH to 10.0 (**b**,**b1**,**b2**).

Figure 5 shows the surface images of the C110 coupons after immersion test in aerated solution at 220 °C for 30 d. It can be seen from Figure 5a,b that the blank sample is covered by corrosion products that render the sample surface tarnished, while the addition of buffer reagents makes the alloy keep its metal luster. The SEM image in Figure 5a1 indicates that, before cleansing of sample surface, the corrosion product on the blank sample is loose and discrete, and many particulate phases exist which are mainly composed of iron oxides (see the EDS data inset). In contrast, with buffer reagents, shown in Figure 5a2, the surface is very clean even without cleansing, suggesting that the sample is slightly corroded. After removing the corrosion products, as shown in Figure 5a2,b2, it is clear that the blank sample’s surface beneath the product layer is notably etched, displaying uniform etching and pitting corrosion features, while the sample under buffer condition is fresh and clean. With regard to the corrosion rates, as given in Figure 5, the qualitative categorization of the corrosion rate of the blank sample (0.3149 mm a^−1^) is “High”, while that of the sample under buffer condition (0.0171 mm a^−1^) is “Low” according to the AMPP NACE SP0775-2023 Standard [23]. Compared with the 30 d immersion corrosion results in Figure 3 and Figure 4, it is evident that the presence of oxygen in the aerated corrosion medium significantly facilitates the dissolution of C110 steel if inhibition measures are not applied. Once the Na_2_CO_3_/NaHCO_3_ buffer reagents are introduced, the corrosion process can be mitigated very effectively, with the inhibition efficiency rising to 94.6% in this case.

**Figure 6:**
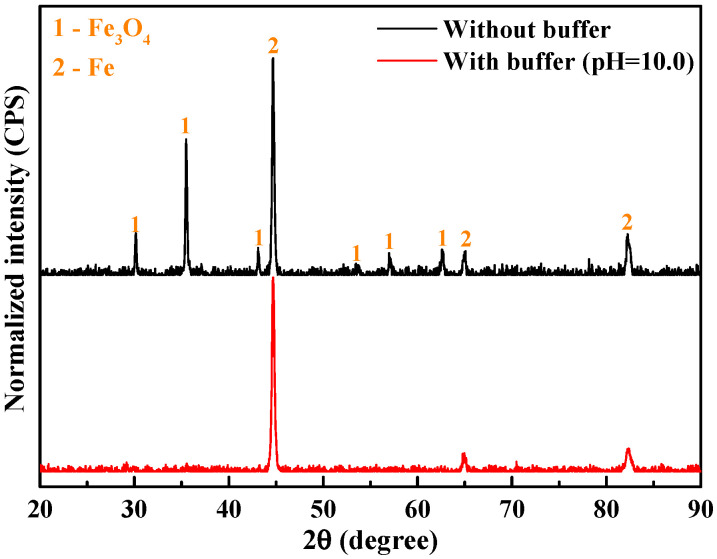
XRD spectra of C110 steel after immersion corrosion at 220 °C for 30 days in aerated NaBr solution without buffer reagents and with buffer reagents adjusting the pH to 10.0.

We employed an XRD approach to analyze the corroded surface before cleansing (see Figure 5), and the spectra are shown in Figure 6. In the absence of buffer reagents, the corrosion product mainly comprises the Fe_3_O_4_ phase, consistent with the literature [19] which suggests that for downhole environments at 150 °C and above, or with high concentration of halide ions [24], the Fe_3_O_4_ phase tends to form. For the sample with buffer reagents, only the iron signals from the steel substrate are detected, indicating that the corrosion product is very thin, probably presenting in the form of nanoscale passive film.

**Figure 7:**
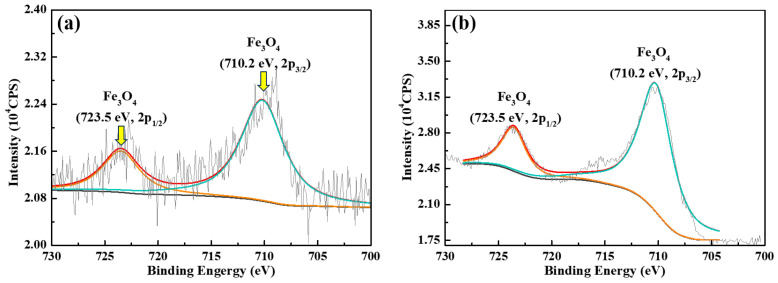
High-resolution XPS spectra of Fe 2p after immersion corrosion at 220 °C for 30 days in aerated NaBr solution. The characterizations were conducted before cleansing of the corrosion products. (**a**) Without buffer; (**b**) with buffer (pH = 10.0).

In order to compare the surface phase composition of the immersed samples with corrosion products, XPS characterizations were performed, and the high-resolution spectra of Fe 2p are shown in Figure 7. It is evident that the surfaces of both samples comprise Fe_3_O_4_, as marked at the binding energies of 710.2 eV (2p_3/2_) and 723.5 eV (2p_1/2_) [25], which is also supported by the XRD result. The peak intensities of the blank sample are much lower than those of the buffer condition, and the blank sample shows more noises. As per the morphologies in Figure 5a1,b1, the noises of the blank sample should be attributed to the complex structure and composition of the thick corrosion products, while the steep peaks for the buffer condition indicates the formation of dense passive film that well protects the steel substrate.

**Figure 8:**
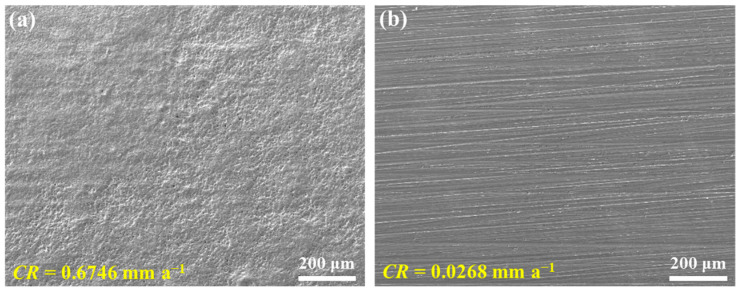
Surface morphologies of C110 steel after immersion corrosion at 220 °C for 7 days in aerated NaBr solution with 0.1 MPa CO_2_ and 0.1 MPa H_2_S in the absence of buffer reagents (**a**) and presence of buffer reagents adjusting the pH to 10.0 (**b**).

Furthermore, considering that CO_2_ and H_2_S may also exist in the downhole environment, 0.1 MPa CO_2_ and 0.01 MPa H_2_S were also added to the corrosion medium for corrosion performance evaluation. Figure 8 shows the corrosion morphologies of C110 coupons after 7 d immersion at 220 °C in aerated NaBr solution with 0.1 MPa CO_2_ + 0.1 MPa H_2_S. It is distinct that the corrosion degree is more severe than the aforementioned samples. In Figure 8a, the surface is severely corroded and with a large number of pitting sites, whereas the sample with buffer reagents still retains the abrasive scratches and no pits can be found, as displayed in Figure 8b. Comparing these with the corrosion rates in Figure 2, it can be seen that the corrosion rate of the blank sample (0.6746 mm a^−1^) is 17 times greater in the aerated CO_2_/H_2_S environment, while in the buffer solution (pH = 10.0), the corrosion rate (0.0268 mm a^−1^) is only 4 times higher than that in the deaerated solution, and the inhibition efficiency is 96.1%. Based on all the immersion corrosion results, it is inferred that the more aggressive medium, the higher inhibition effect of Na_2_CO_3_/NaHCO_3_ buffer reagents in the NaBr annulus protection fluid environment at 220 °C.

### 3.3. Electrochemical Corrosion Measurements

The immersion corrosion results evidence the notable inhibition effect of Na_2_CO_3_/NaHCO_3_ buffer reagents on the corrosion of C110 steel in NaBr annulus protection fluid environment. To analyze the underlying tuning mechanism of buffer reagents, we carried out potentiodynamic polarization (PD) measurements in aerated solution at 25–85 °C. It should be noted that although we are unable to conduct an electrochemical test at 220 °C due to the apparatus limitation, the PD data can still reflect the function of buffer reagents with the increase in temperature.

**Figure 9:**
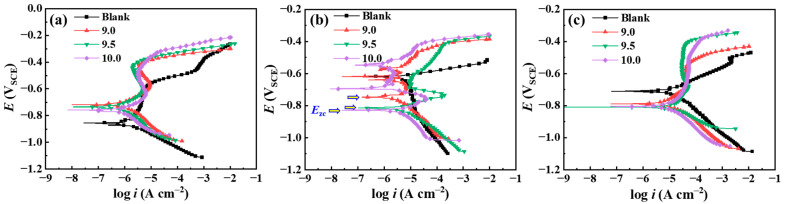
Potentiodynamic polarization curves of C110 steel in aerated NaBr solution without and with buffer reagents at different temperatures. (**a**) 25 °C; (**b**) 50 °C; (**c**) 85 °C.

**Figure 10:**
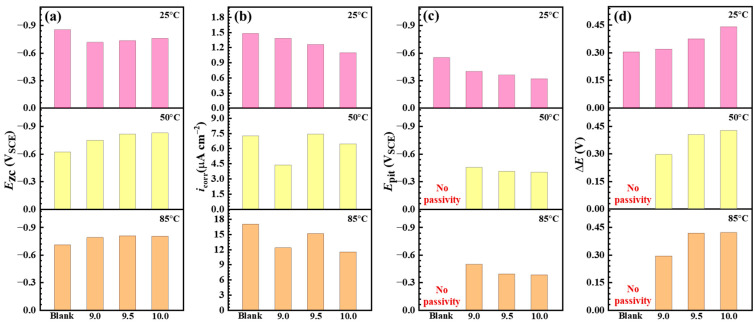
Electrochemical parameters acquired by fitting the potentiodynamic polarization curves. (**a**) Zero-current potential (*E*_zc_); (**b**) self-corrosion current density (*i*_corr_); (**c**) pitting potential (*E*_pit_); (**d**) width of passive region (Δ*E*).

Figure 9 shows the PD curves of C110 steel in aerated NaBr solution without and with buffer reagents at 25, 50, and 85 °C, and the corresponding fitting results are given in Table 2 and Figure 10. The *E*_zc_ stands for zero-current potential, which is generally equal to the corrosion potential [26]; *i*_corr_ is the self-corrosion current at *E*_zc_; *E*_pit_ is the pitting (or transpassive) potential; and Δ*E* is the width of the passive region (Δ*E* = *E*_pit_ − *E*_zc_). In Figure 9a, *E*_zc_ and *E*_pit_ are increased by adding buffer reagents, e.g., Δ*E* increases from 0.304 to 0.440 V, indicating that the anodic dissolution reactions are inhibited at 25 °C and the passivity of C110 steel is enhanced. With an increase in the temperature to 50 and 85 °C, seen in Figure 9b,c, *E*_zc_ in the buffer condition becomes lower than in the blank sample, suggesting that the inhibition mechanism converts to cathodic type at higher temperatures. The *E*_pit_ values at 50 and 85 °C remain high in the presence of buffer reagents, with the most positive *E*_pit_ values of −0.319 and −0.402 V_SCE_ at pH = 10.0, while the blank sample shows active dissolution (no passivity) behaviors at increased temperatures One interesting phenomenon is found in Figure 9b, where in the presence of buffer reagents, the PD curves above *E*_zc_ exhibit several “pseudo” zero-current potentials. Song et al. [27] also reported this feature in high-pH (9.5) corrosion of X100 pipeline steel in carbonate/ bicarbonate solution under the impact of tensile stress, and they suggested that the emergence of these “pseudo” zero-current potentials is because of the multiple anodic reactions. Therefore, we believe the reason that this phenomenon only exists for the 50 °C condition should be due to the inhibition mode switching from anodic to cathodic at this temperature.

Figure 10 compares the values of *E*_zc_, *i*_corr_, *E*_pit_, and Δ*E* as a function of buffered pH at different temperatures. Note that the y-axis of *E*_zc_ and *E*_pit_ are plotted to the negative direction to better display the potential values. In the presence of buffer reagents, *E*_zc_ gradually decreases with the pH (Figure 10a), while *E*_pit_ shifts to the positive direction (Figure 10c), leading to the enhanced Δ*E* in the three temperature conditions (Figure 10d). This indicates that the addition of buffer reagents can provide stronger passivation capability to C110 steel, enabling the formation of stable passive film that protects the substrate from active dissolution. *i*_corr_ notably increases with the temperature (Figure 10b), from nearly 1 μA cm^−2^ to greater than 11 μA cm^−2^, but it does not show a clear variation trend with the increase in pH. In addition, one may find that we marked “No passivity” for the blank samples in the charts of 50 and 85 °C, as evidenced by the PD curves. It can be inferred that at even higher temperatures, e.g., 220 °C, the favored anodic dissolution reaction will also render the steel surface unable to reach the passivation state in the absence of buffer reagents, which is the primary reason for its more severe corrosion.

**Figure 11:**
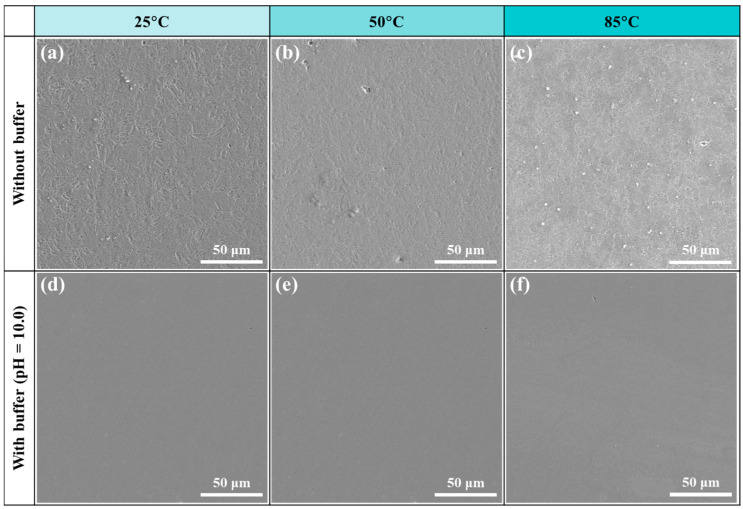
Corrosion morphologies of C110 steel after potentiodynamic polarizations at different temperatures. (**a**–**c**) Aerated NaBr solution without buffer reagents; (**d**–**f**) aerated NaBr solution with buffer reagents (pH = 10.0).

Furthermore, we also observed the surface corrosion morphologies after the PD tests, as shown in Figure 11. In the absence of buffer reagents, the samples are distinctly corroded, revealing the metallurgical microstructures as well as the corrosion pits due to anodic dissolution. With the increase in temperature, the pitting corrosion features are more noticeable, particularly at 85 °C, and a large number of pits can be found. In contrast, the corrosion degree is significantly lower in the presence of buffer reagents (pH = 10.0), again evidencing the noteworthy inhibition effect of the Na_2_CO_3_/NaHCO_3_ buffer system in the corrosion protection of C110 steel.

## 4. Discussion

The experimental results reveal that the Na_2_CO_3_/NaHCO_3_ buffer reagents effectively inhibit the corrosion of C110 steel in concentrated NaBr solution for annulus protection at 220 °C. Compared with the deaerated solution, the aerated condition is more aggressive, leading to the severe corrosion (the corrosion rate is classified as “High”). However, once the buffer system is introduced, the corrosion rate is notably decreased, and the highest inhibition efficiency reaches 96.1% in the presence of CO_2_/H_2_S. We now discuss the underlying corrosion and inhibition mechanism.

### 4.1. Corrosion in the Absence of Buffer Reagents

In the neutral NaBr solution without Na_2_CO_3_/NaHCO_3_ buffer reagents, the anodic dissolution produces Fe^2+^ ions, and the process is controlled by hydrogen evolution (deaerated solution) and oxygen reduction (aerated solution) reactions below:

2H_2_O + 2e → H_2_ + 2OH^−^ (deaerated) (2)

O_2_ + 2H_2_O + 4e → 4OH^−^ (aerated) (3)

**Figure 12:**
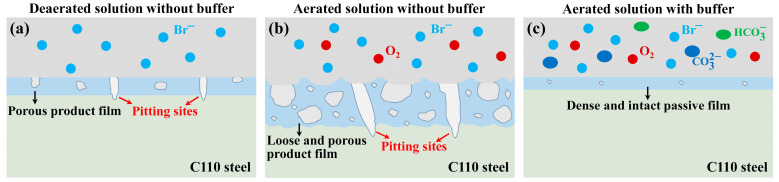
Schematic diagrams of the corrosion inhibition mechanism of NaBr solution with NaCO_3_/NaHCO_3_ buffer reagents.

Reactions (2) and (3) lead to the formation of OH^−^ which tends to combine with Fe^2+^ to generate Fe(OH)_2_ gels [24] and precipitate on the steel surface. As is well known, oxygen reduction has a greater capability of accelerating the anodic dissolution of Fe [28], and this is the reason for the findings in Figure 5a1, i.e., the 30 d immersion in aerated condition, which exhibits much higher degree of corrosion than that in Figure 3a in the deaerated condition. Since oxygen reduction is diffusion controlled, the limiting diffusive current of cathodic reaction will promote the oxidation of Fe(OH)_2_ to Fe_3_O_4_ via Reaction (4) [29], which explains the presence of Fe_3_O_4_ in XRD and XPS spectra. Meanwhile, our experimental results indicate that the Fe(OH)_2_ to Fe_3_O_4_ transformation leads to the formation of loose and porous corrosion products (see Figure 5a1), as is depicted in Figure 12.

3Fe(OH)_2_ + 1/2O_2_ → Fe_3_O_4_ + 3H_2_O (4)

Furthermore, when 0.1 MPa CO_2_ and 0.1 MPa H_2_S are added to the aerated corrosion medium, although with low partial pressure (the total pressure is 10 MPa), the gaseous phases dissolve into the solution and induce the following reactions [30,31]:

CO_2_ + H_2_O → H_2_CO_3_ (5)

(6) $$H_{2}{\mathrm{CO}}_{3}\;\to \;H^{+}+{\mathrm{HCO}}_{3}^{-}$$

(7) $${\mathrm{HCO}}_{3}^{-}\to \;H^{+}+{\mathrm{CO}}_{3}^{2-}$$

H_2_S + H_2_O → H_3_O^+^ + HS^−^ (8)

HS^−^ + H_2_O → H_3_O^+^ + S^2−^ (9)

Clearly, Reactions (5) to (9) induce sweet corrosion which lowers the pH, favoring Reaction (3), and facilitate the severe corrosion of C110 steel. Meanwhile, the dissolving reactions of CO_2_ and H_2_S should also lead to the generation of FeCO_3_ and Fe_x_S_y_ (due to the complex state of sulfide ions) as part of the corrosion products; however, they are not analyzed in detail since this is not the core issue of the present study.

### 4.2. Na_2_CO_3_/NaHCO_3_ Induced Passivation

When the Na_2_CO_3_/NaHCO_3_ buffer system is introduced into the NaBr solution, in addition to Reaction (7), the reversed reaction may also take place:

(10) $${\mathrm{CO}}_{3}^{2-}+H_{2}O\;\to \;{\mathrm{OH}}^{-}+{\mathrm{HCO}}_{3}^{-}\;(\mathrm{alkaline})$$

Many works have shown that FeCO_3_ will form in ${\mathrm{CO}}_{3}^{2-}$ and ${\mathrm{HCO}}_{3}^{-}$ solutions via the following reactions (see Ref. [30] and the cited papers therein):

(11) $$\mathrm{Fe}2^{+}+{\mathrm{CO}}_{3}^{2-}\;\to \;{\mathrm{FeCO}}_{3}$$

(12) $$\mathrm{Fe}2^{+}+2{\mathrm{HCO}}_{3}^{-}\;\to \;{\mathrm{Fe}({\mathrm{HCO}}_{3})}_{2}$$

Fe(HCO_3_)_2_ → FeCO_3_ + H_2_O +CO_2_ (13)

However, it should be noted that few works have been conducted at elevated temperatures, e.g., above 200 °C, until Tanupabrungsun and Nešić [32] produced a systematic study on an Fe-CO_2_-H_2_O system at a wide temperature range (25 to 250 °C). Importantly, they found, theoretically (by constructing Pourbaix diagram) and experimentally (immersion corrosion), that Fe_3_O_4_ is the sole phase at 200–250 °C, while below 200 °C FeCO_3_ is the dominant phase of the corrosion products. This is in good agreement with our XRD data in Figure 6. Similar comments were given in a previous review article [19], despite the fact that the FeCO_3_ to Fe_3_O_4_ transformation generally takes place at 150 °C and above. Moreover, it was revealed that the morphology of the Fe_3_O_4_ layer is denser and thinner but more protective, leading to a notable decrease in corrosion rate [32], which is in good agreement with our results in Figure 5 and Figure 7.

According to the surface morphology in Figure 5b1, XRD data in Figure 6, and the PD results in Figure 9, there is no doubt that the Fe_3_O_4_ phase, in the presence of buffer reagents at 220 °C, exists in the form of passive film rather than the corrosion product layer due to the rapid formation of Fe_3_O_4_ passive film as the barrier layer and limited dissolution of the steel substrate. Owing to the superior blocking effect of the passive film, the corrosion rate declines significantly. Certainly, the excellent inhibition efficiency of the passive film should also be a result of the application of less aggressive Br^−^ ions [33] instead of Cl^−^ as the annulus protection fluid.

### 4.3. Corrosion Product vs. Passive Film

Based on the experimental results and discussions, it is clear that without buffer reagents, the steel surface is covered by corrosion products, while dense and intact passive film forms in the presence of buffer reagents, as schematically depicted in Figure 12. In the deaerated solution which only contains NaBr, seen in Figure 12a, the dissolution of steel substrate leads to the formation of porous product film. The pores act as the diffusion channels of ions, resulting in the small pitting sites. In Figure 12b, the aerated solution is more corrosive due to the presence of dissolved O_2_. The cathodic oxygen reduction reaction facilitates the anodic dissolution of Fe, forming much thicker but more loose and porous product film with a lower protection ability for the substrate. As a consequence, the steel surface is severely etched, and the channels render the generation of larger and deeper corrosion pits. In contrast, when the buffer reagents are added, as shown in Figure 12c, a thinner but denser and more protective passive film of Fe_3_O_4_ is generated. Although there could be point defects inside the passive film [34], the mass diffusions in the barrier layer is significantly lower than that in the porous product film, thus contributing to the notably declined corrosion rate under the buffer condition.

## 5. Conclusions

In the present work, the Na_2_CO_3_/NaHCO_3_ buffer system is employed to mitigate the corrosion of C110 steel in 39.4 wt% NaBr annulus protection fluid at 220 °C. Immersion tests, surface morphology characterizations, XRD, XPS, and electrochemical analyses were conducted to analyze the inhibition effect of the buffer reagents. The main conclusions are summarized below:

(1) The Na_2_CO_3_/NaHCO_3_ buffer reagents effectively inhibit the corrosion of C110 steel at 220 °C. Compared with the deaerated solution, the aerated condition is more aggressive and induces severe corrosion, and the highest corrosion rate can reach 0.6746 mm a^−1^. However, once the buffer system is introduced, the corrosion rate is notably decreased, and the highest inhibition effect is obtained in aerated solution with CO_2_/H_2_S.
(2) Under the buffered condition, a higher pH leads to better inhibition performance. Meanwhile, the mitigation effect of the Na_2_CO_3_/NaHCO_3_ buffer system is more pronounced in the corrosion medium with higher aggressivity, i.e., the inhibition efficiency lies in the order of deaerated solution (82.4%) < aerated solution (94.6%) < aerated solution with CO_2_/H_2_S (96.1%).
(3) In the absence of buffer reagents, loose and porous corrosion products (mainly Fe_3_O_4_) are generated with insufficient protection to the steel substrate, resulting in uniform etching and pitting corrosion. The addition of buffer reagents facilitates the formation of thinner but denser and more protective Fe_3_O_4_ passive film, which is the primary reason for the high inhibition efficiency.

## Figures and Tables

**Table 1:** The chemical composition of C110 steel (wt%).

Element	C	Si	Mn	Ni	Cr	Mo	Cu	P	S	Fe
Content	0.27	0.17	0.44	0.06	0.47	0.82	0.04	0.007	0.001	Bal.

**Table 2:** Electrochemical parameters acquired by fitting the potentiodynamic polarization curves.

Temperature	Condition	*E*_zc_ (V_SCE_)	*i*_corr_ (µA cm^−2^)	*E*_pit_ (V_SCE_)	Δ*E* (V)
25 °C	Blank	−0.855	1.479	−0.551	0.304
	9.0	−0.717	1.380	−0.398	0.319
	9.5	−0.733	1.259	−0.359	0.374
	10.0	−0.759	1.096	−0.319	0.440
50 °C	Blank	−0.621	7.244	No passivity	No passivity
	9.0	−0.748	4.365	−0.452	0.296
	9.5	−0.814	7.413	−0.410	0.404
	10.0	−0.829	6.457	−0.402	0.427
85 °C	Blank	−0.710	16.982	No passivity	No passivity
	9.0	−0.789	12.303	−0.496	0.293
	9.5	−0.808	15.136	−0.390	0.418
	10.0	−0.802	11.482	−0.381	0.421

## Data Availability

The data that support the findings of this study are available from the corresponding author upon reasonable request.
